# Predicting potential and quality distribution of *Anisodus tanguticus* (Maxim.) Pascher under different climatic conditions in the Qinghai–Tibet plateau

**DOI:** 10.3389/fpls.2024.1369641

**Published:** 2024-06-03

**Authors:** Chen Chen, Bo Wang, Jianan Li, Yuanming Xiao, Kaiyang Chen, Na Liu, Guoying Zhou

**Affiliations:** ^1^ Anhui Provincial Engineering Laboratory for Efficient Utilization of Featured Resource Plants, College of Life Sciences, Huaibei Normal University, Huaibei, Anhui, China; ^2^ Chinese Academy of Sciences Key Laboratory of Tibetan Medicine Research, Northwest Institute of Plateau Biology, Xining, China

**Keywords:** medicinal plant, maximum entropy model, distribution area, Qinghai-Tibet Plateau, *A. tanguticus*

## Abstract

*Anisodus tanguticus* (Maxim.) Pascher, a distinctive medicinal plant native to the Qinghai-Tibet Plateau, China, has garnered attention due to increasing market demand. This study explores the impact of environmental factors on the distribution and levels of active compounds namely anisodamine, anisodine, and atropine within *A. tanguticus*. Our goal was to identify suitable cultivation areas for this plant. This study employs the maximum entropy model to simulate the suitable area of *A. tanguticus* under current conditions and three climate change scenarios during the 2050s and 2070s. The finding revealed that altitude, precipitation in the warmest season (Bio 18), the average annual temperature (Bio 1) exerted significant influences on the distribution of *A. tanguticus*. Among the environmental factors considered, temperature difference between day and night (Bio 2) had the most substantial impact on the distribution of anisodamine, temperature seasonal variation variance (Bio 4) predominantly influenced anisodine distribution, and Bio 1 had the greatest effected on the distribution of atropine. The suitable areas primarily exist in the eastern Qinghai-Tibet Plateau in China, encompassing a total area of 30.78 × 10^4^ km^2^. Under the climate scenarios for the future, the suitable areas exhibit increasing trends of approximately 30.2%, 30.3%, and 39.8% by the 2050s, and 25.1%, 48.8%, and 60.1% by the 2070s. This research would provide theoretical suggestions for the protection, and cultivation management of *A. tanguticus* resources to face the challenge of global climate change.

## 1 Introduction

Climate is the most important environmental factor affecting species distribution on a regional and global scale. The accelerated development of human civilization during the 20th century, driven by the industrial revolution, has resulted in significant challenges for the ecological environment. These challenges include climate warming and severe environmental damage (Wei and Qiao, 2017). Global warming is already widely recognized, and the warming trend is becoming increasingly obvious. According to the Fifth Assessment Report from the Intergovernmental Panel on Climate Change (IPCC), global temperatures have increased by almost 1°C in the last century. Projections indicate that global surface temperatures are expected to further warm between 1.4°C and 5.8°C by 2100. Species’ responses to warming often manifest themselves as extinctions, adaptations, and migrations (Parmesan and Yohe, 2003; Root et al., 2003). Therefore, forecasting the influence of climate change on the potential geographical distribution of plants has become a central area of biogeography and global change research.

*Anisodus tanguticus* (Maxim.) Pascher, a member of the Solanaceae family, is a significant medicinal plant endemic to the Qinghai-Tibet Plateau in China. It is primarily found in Qinghai, Sichuan, Gansu, and Tibet, thriving on grasslands, riverbanks, mountains, and shrublands (Jin et al., 2020). In the “List of Wild Plants under National Key Protection” approved on August, 1999, *A. tanguticus* was listed as a national second-level key protected plant. Also, it is on the International Union for Conservation of Nature (IUCN) Red List of Threatened Species (Wan et al., 2016). *A. tanguticus* has garnered growing interest from pharmaceutical companies due to its role as a source of tropane alkaloids like anisodine, anisodamine, and atropine. These alkaloids find applications in analgesia, anesthesia, antispasmodics, anti-motion sickness, and the treatment of Parkinson’s disease (Qiu et al., 2021; Zhang et al., 2022; Chen et al., 2022b, c). Wild digging and climate change will exacerbate the loss of the endangered plant habitat. Luckly, progress has been made in the cultivating *A. tanguticus* and efficient fertilization schemes have been established. However, studies of suitable growth zones and the distribution zones of alkaloids are still lacking (Liu et al., 2022b; Chen et al., 2023).

Species distribution models (SDMs) are widely used in conservation biology, invasion biology, and other studies involving species distribution and genetic diversity (Alkhalifah et al., 2022; Yang et al., 2022; Liu et al., 2022a). Currently, species distribution models are widely used, including generalized linear model (GLM), random forest model (RF), ecological niche factor analysis model (ENFA), bioclimate analysis system (BIOCLM) and maximum entropy model (MaxEnt), etc (Pan et al., 2020). Among them, the MaxEnt model has better prediction ability compared with other models, and the size of the sample has little impact on the accuracy of the MaxEnt model in predicting species spatial distribution, so it is widely used in species Potential distribution area prediction (Zheng et al., 2022). Currently, the MaxEnt model is used to simulate the distribution of various endangered plant species on the Tibetan Plateau, such as *Pomatosace filicula*, *Morina kokonorica*, *Morina chinensis*, *Gymnadenia orchidis*, *Gentiana lhassica*, *Rhodiola alterna*, *Lomatogoniopsis alpina*, *Fritillaria unibracteata*, *Fritillaria przewalskii*, *Rheum tanguticum*, *Arenaria brevipetala*, *Fritillaria delavayi*, *Notopterygium incisum*, *Aconitum brunneum*, *Rhododendron anthopogonoides* and *Androsace elatior* (Chen et al., 2022d; Yang et al., 2024; Yuan et al., 2024). Therefore, analyzing the impact of climate change on the suitable areas of *A. tanguticus* has significance for understanding the geographical distribution and quality area of the species. This analysis is beneficial for devising management strategies to adapt to climate change and enhance the protection of *A. tanguticus*.

Our initial hypotheses posited that environmental factors would play a critical role in determining the distribution and quality of *A. tanguticus*, based on the plant’s known ecological preferences and the physiological impact of these factors on plant growth. The study focused on *A. tanguticus*, presently endangered in China. Utilizing data collection and field investigation, we employed the Maxent model, ArcGIS software, and statistical analysis to elucidate the connection between geographical distribution and environmental variables. On this basis, the suitable regions and alkaloid quality regions of *A. tanguticus* in three climate scenarios under current climatic conditions and future climatic conditions (2050s and 2070s) were predicted. This study is a foundation for selecting sites for the artificial planting and cultivation of *A. tanguticus*. Additionally, it holds significance for safeguarding wild resources and screening excellent germplasm resources.

## 2 Methods

### 2.1 Geographic distribution information

The distribution data is derived from surveys conducted at 71 sample sites by our team, along with searches conducted on the Chinese Plant Digital Herbarium (https://www.cvh.ac.cn/), NSII Chinese Herbarium Resource Platform (http://www.nsii.org.cn/2017/home.php), and CNKI Database (http://www.cnki.net/). Through these platforms, we specifically searched for the keyword “*A. tanguticus* species”, meticulously verifying and recording their latitude and longitude information. When a record was missing precise geographic coordinates, we used Google Earth (http://ditu.google.cn) to determine the latitude and longitude based on the geographic location described. This comprehensive process yielded the distribution points of *A. tanguticus* in China, following the careful exclusion of erroneous and duplicate data. Collectively, these sources served as the foundation for the initial organization of distribution data, culminating in the collection of a total of 260 distribution records (**Supplementary Figure 1**). Subsequently, we exported this distribution data from Excel and converted it into CSV format to facilitate its utilization in Maxent modeling.

### 2.2 Environment variable collection

The current climate data (1970s-2000s) and future climate data (2050s, 2070s) used in this study were downloaded from the WorldClim database (http://www.worldclim.org) at a spatial resolution of 30 arc-seconds (approximately 1 km). This dataset includes 19 climate variables (Bio 1-Bio 19) associated with temperature and precipitation. The Coupled Model Intercomparison Project Phase 5 produced four Representative Concentration Pathways (RCPs): RCP 2.6, 4.5, 6.0, and 8.5. These pathways are established on radiative forcing, contingent upon CO_2_ emissions. RCP2.6 and RCP8.5 are low and high CO_2_ emission scenario whereas intermediate emission scenarios are RCP4.5 and RCP6.0. In this study, RCP2.6, RCP6.0, and RCP8.5 emission scenarios have been used. Elevation variables also downloaded from WorldClim at a spatial resolution of 30 arc-seconds. Human Footprint Index is sourced from the Columbia University Center for International Earth Science Information Network (CIESIN) database (https://sedac.ciesin.columbia.edu/data/set/wildareas-v2-human-footprint-geographic).

Soil factor data including available potassium (AK), total phosphorus (TP), available phosphorus (AP), total potassium (TK), available nitrogen (AN), total nitrogen (TN), soil organic matter (SOM) and soil pH were collected from the National Tibetan Plateau Data Center (https://data.tpdc.ac.cn/home). The base map of China was obtained from the National Basic Geographic Information System (http://nfgis.nsdi.gov.cn/). We resampled the 8 soil variables and human activity variables at a spatial resolution of 30 arc-seconds.

In order to eliminate the problem of multicollinearity between environmental factors, we calculated the Pearson correlation coefficients between the 29 environmental factors using EMTools software. For variables with correlation coefficients greater than 0.8, we retained variables of high importance to the model and deleted variables of low importance. Variables with 0 contribution were also removed when constructing the model.

### 2.3 Prediction of suitable areas

We imported the files containing 260 distribution points of *A. tanguticus* and the above-mentioned environmental variables into the MaxEnt software. The MaxEnt parameter settings as 75% of the data is used for a training set for model building, 25% is used for model testing, and the simulation was repeated 10 times (Chen et al., 2022d). Maximum number of iterations is 5 000. Other parameters use MaxEnt software default settings. The “10 percentile training presence logistic threshold” was used as a threshold to classify non-suitable (0–0.3076) and suitable (0.3076–1) habitats for species distribution, and the suitable habitats were equally divided into three equal parts: low suitable (0.3076–0.5384), moderately suitable (0.5384–0.7692), and highly suitable (0.7692–1) (Wan et al., 2021). The area under the ROC curve (AUC value) was used to evaluate the predictive accuracy. The range of AUC is from 0 to 1, and the higher the AUC value, the more accurate it is. The model performance was classified as failed (0.5–0.6), poor (0.6–0.7), average (0.7–0.8), good (0.8–0.9) or excellent (0.9–1.0). We constructed a modern niche model and projected it onto three Representative Concentration Pathways (RCP2.6, RCP6.0, and RCP8.5) for the 2050s and 2070s, employing the optimized parameter combination mentioned above. Subsequently, we utilized ArcGIS software to reclassify and visually represent the results, categorizing them into four levels based on the distribution rate (Wang et al., 2021).

### 2.4 Regional suitability for high-quality

In the study the quality zoning of the *A. tanguticus* samples was collected with a total of 71 samples. According to the research method of predecessors, we quantified the content of three tropine alkaloids (anisodamine, anisodine, and atropine) in *A. tanguticus* (Chen et al., 2022b). Using anisodine, anisodamine and atropine as dependent variables and 28 environmental factors as independent variables, multiple regression analysis was performed to establish a regression model between anisodine, anisodamine, atropine and environmental factors. Use the stepwise regression method for variable selection. The smaller the root mean square error (RMSE) and mean absolute error (MAE) values of the model, the better the results. The spatial analysis tools in ArcGIS10.4 were used, combined with the above model results, to predict the spatial distribution of anisodine, anisodamine and atropine contents in the current climatic conditions and future climatic conditions (Gao et al., 2022).

## 3 Results

### 3.1 Dominant environmental variables and their response curves

After the MaxEnt model of *A. tanguticus* in the Qinghai-Tibet Plateau was established, the AUC value of the validation data was 0.975. This suggests a strong correlation between the most significant environmental variable selected for prediction and the geographical distribution, affirming the reliability of the prediction conclusion. Consequently, the MaxEnt model proves suitable for predicting the potential distribution area of *A. tanguticus*.

Choosing environmental factors is crucial for assessing modeling accuracy. Evaluating these factors based on the knife cutting method results (**Supplementary Figure 2**) and the environmental contribution rate (**Supplementary Table 1**) is necessary to identify the dominant factors affecting the current potential geographic distribution of *A. tanguticus*. By participating in modeling the contribution rate of environmental factors, the important values of displacement, the knife cut plot, and the main parameters of environmental factors are comprehensively considered. The analysis showed that the main environmental factors affecting the distribution of *A. tanguticus* were altitude (Alt), precipitation in the warmest season (Bio 18), average annual temperature (Bio 1), range of annual temperature changes (Bio 7), human activities, variance of seasonal variation in precipitation (Bio 15), the ratio of the temperature difference between day and night to the annual temperature difference (Bio 3).

**Supplementary Figure 3** illustrates the suitable range of dominant environmental factors influencing the potential distribution of *A. tanguticus*. All seven response curves exhibit a unimodal and approximately normal distribution, suggesting that the predictions of the model in this study are more accurate. When the distribution probability is greater than 0.5, the corresponding ecological factor value is suitable for the growth of species. According to the response curve of environmental factors, Alt (3000~4400 m), Bio 18 (200~380 mm), Bio1 (0~4.5°C), Bio 7 (33~38 °C), human activities (10~26), Bio 15 (85~100 mm), and Bio 3 (38%~46%) are suitable for the growth of *A. tanguticus*.

### 3.2 Prediction of suitable areas

**Figure 1:**
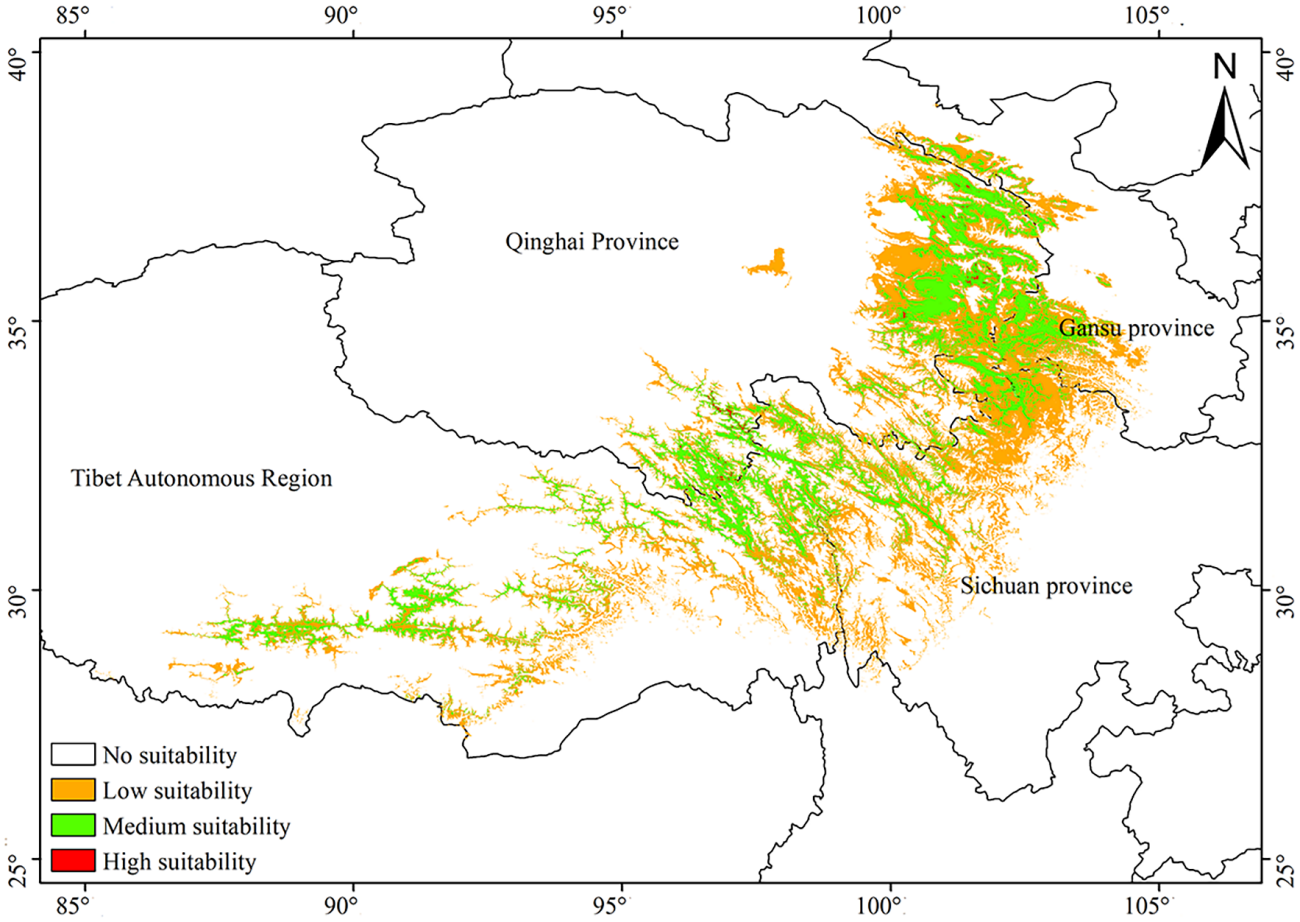
Distribution pattern of *A. tanguticus* under the current climate.

We utilized MaxEnt models and ArcGIS software to forecast the potential distribution of *A. tanguticus* under the current climate. Employing the natural break point method, we categorized the potential distribution into four suitability grades: the cutoff value is 0–0.3076 as the non-suitable zone, 0.3076–0.5384 is a low suitable area, 0.5384–0.7692 is the moderately suitable area, and 0.7692–1 is a highly suitable area. As shown in **Figure 1**, a highly suitable area was concentrated in the Qilian Mountain and Guoluo Prefecture in Qinghai Province. The calculated areas indicated that the highly suitable area covered 0.08 × 10^4^ km^2^, the area of medium suitable area was 9.63 × 10^4^ km^2^, and the area of the low suitable area was 21.04 × 10^4^ km^2^, accounting for 0.01%, 1.00% and 2.18% of the total area in China, respectively.

**Figure 2:**
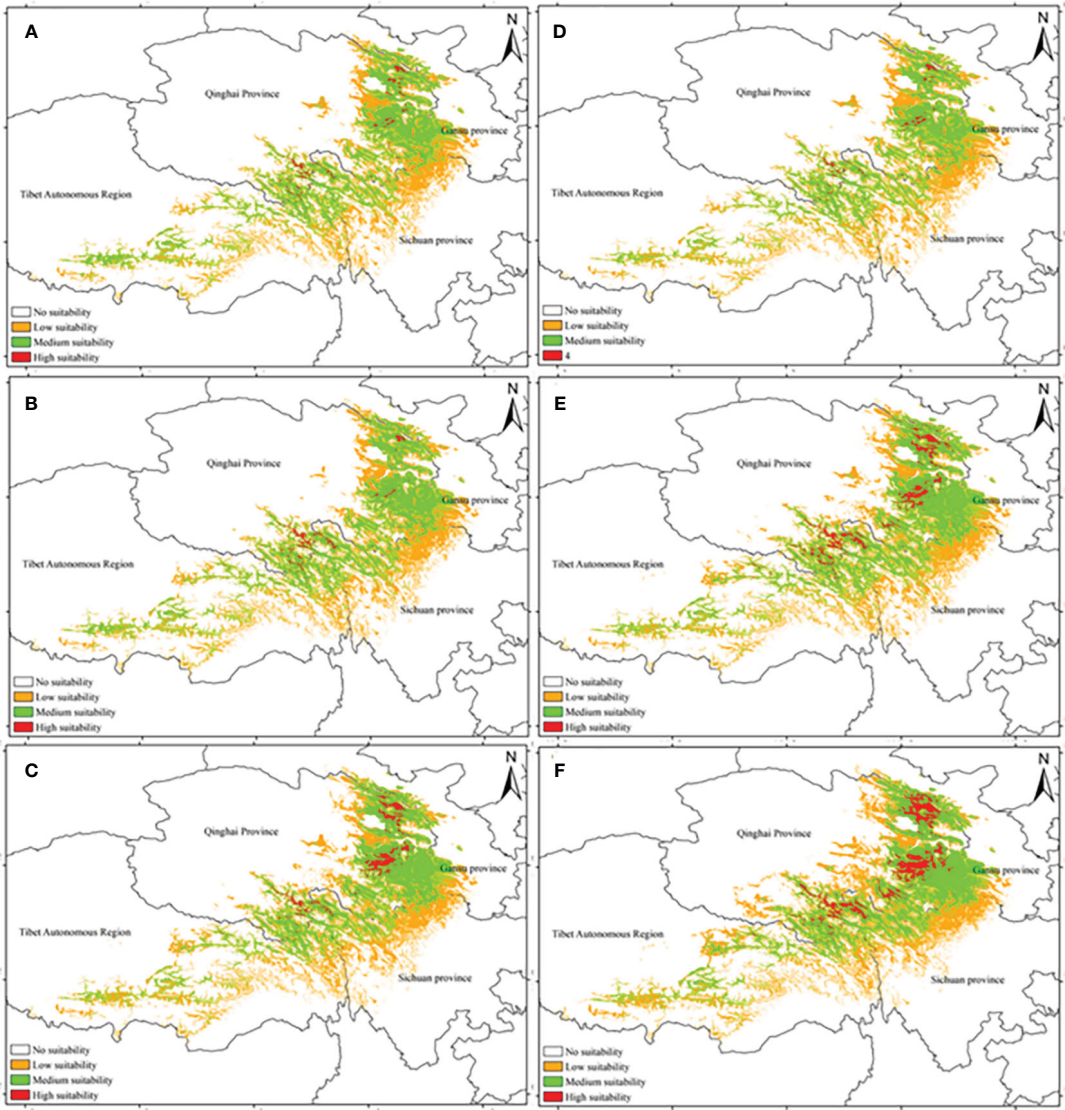
Distribution pattern of *A*. *tanguticus* under three climatic scenarios in the 2050s and 2070s [**(A)** the RCP2.6 scenario in the 2050s; **(B)** the RCP6.0 scenario in the 2050s; **(C)** the RCP8.5 scenario in the 2050s; **(D)** the RCP2.6 scenario in the 2070s; **(E)** the RCP2.6 scenario in the 2070s; **(F)** the RCP2.6 scenario in the 2070s].

**Figure 2** displays the potential distribution of *A. tanguticus* under three RCPs in the 2050s and 2070s. Based on the area calculations, the highly suitable area covered 0.81 × 10^4^ km^2^, the moderately suitable area covered 15.87 × 10^4^ km^2^, and the low suitable area extended to 23.40 × 10^4^ km^2^. These areas represented 0.08%, 1.65%, and 2.43% of the total area in China, respectively, under the RCP2.6 scenario (**Figure 2A**). According to the area calculation, the area of the highly suitable area was 0.79 × 10^4^ km^2^, the area of the medium suitable area was 16.01× 10^4^ km^2^, and the area of the low suitable area was 23.30× 10^4^ km^2^, accounting for 0.08%, 1.67% and 2.42% of the total area in China, respectively, in the RCP6.0 scenario (**Figure 2B**). Based on area calculations, the highly suitable area covered 1.88 × 10^4^ km^2^, the moderately suitable area spanned 16.01 × 10^4^ km^2^, and the low suitable area extended to 23.30 × 10^4^ km^2^. These areas accounted for 0.20%, 1.74%, and 2.53% of the total area in China, respectively, under the RCP8.5 scenario (**Figure 2C**).

According to the area calculation, the highly suitable area was 0.62 × 10^4^ km^2^, the medium suitable area was 14.65× 10^4^ km^2^, and the area of the low suitable area was 23.25× 10^4^ km^2^. These areas accounted for 0.06%, 1.52%, and 2.41% of the total area in China, respectively, under the RCP2.6 scenario (**Figure 2D**). The highly suitable area was 2.44 × 10^4^ km^2^, the medium suitable area was 18.88× 10^4^ km^2^, the low suitable area was 24.48× 10^4^ km^2^, accounting for 0.03%, 1.96% and 2.54% of the total area in China, respectively, in the RCP6.0 scenario (**Figure 2E**). Moreover, the highly suitable area was 3.74 × 10^4^ km^2^, the medium suitable area was 18.92× 10^4^ km^2^, and the low suitable area was 26.64× 10^4^ km^2^, accounting for 0.39%, 1.96% and 2.76% of the total area in China, respectively in the RCP8.5 scenario (**Figure 2F**).

### 3.3 Prediction of regional suitability for high-quality suitable areas

The equations showing the relationship between the tropine alkaloids contents in *A. tanguticus* and the main ecological factors were:


$$\text{Y}1=3.993-0.022\text{X}1\;({\text{R}}^{2}=0.11,\;P<0.05,\;\text{Y}1=\text{anisodamine},\;\text{X}1=\text{Bio}\;2)$$

$$\begin{array}{l} \text{Y}2=7.5720-0.0004\;\text{X}1-0.009\;\text{X}2+0.0346\;\text{X}3-0.0373\;\text{X}4+0.0550\;\text{X}5-\\ 1.6150\;\text{X}6\;({\text{R}}^{2}=0.39,\;P<0.05,\;\text{Y}2=\text{anisodine},\;\text{X}1=\text{Bio}\;4,\;\text{X}2=\text{Bio}\;12,\\ \;\text{X}3=\text{Bio}\;13,\;\text{X}4=\text{Bio}15,\;\text{X}5=\text{SOM},\;\text{X}6=\text{TN}) \end{array}$$

$$\begin{array}{l} \text{Y}3=24.0211-0.1347\;\text{X}1+0.071\;\text{X}2+0.1128\;\text{X}3-0.0442\;\text{X}4-0.0448\;\text{X}5\\ \;({\text{R}}^{2}=0.67,\;P<0.05,\;\text{Y}3=\text{atropine},\;\text{X}1=\text{Bio}\;1,\;\text{X}2=\text{Bio}\;2,\;\text{X}3=\;\text{Bio}\;6,\;\\ \text{X}4=\text{Bio}\;13,\;\text{X}5=\text{Bio}15) \end{array}$$

Significant linear relationships exist between three tropine alkaloids and environmental factors. This information can be employed to predict the suitable area of tropine alkaloids.

**Figure 3:**
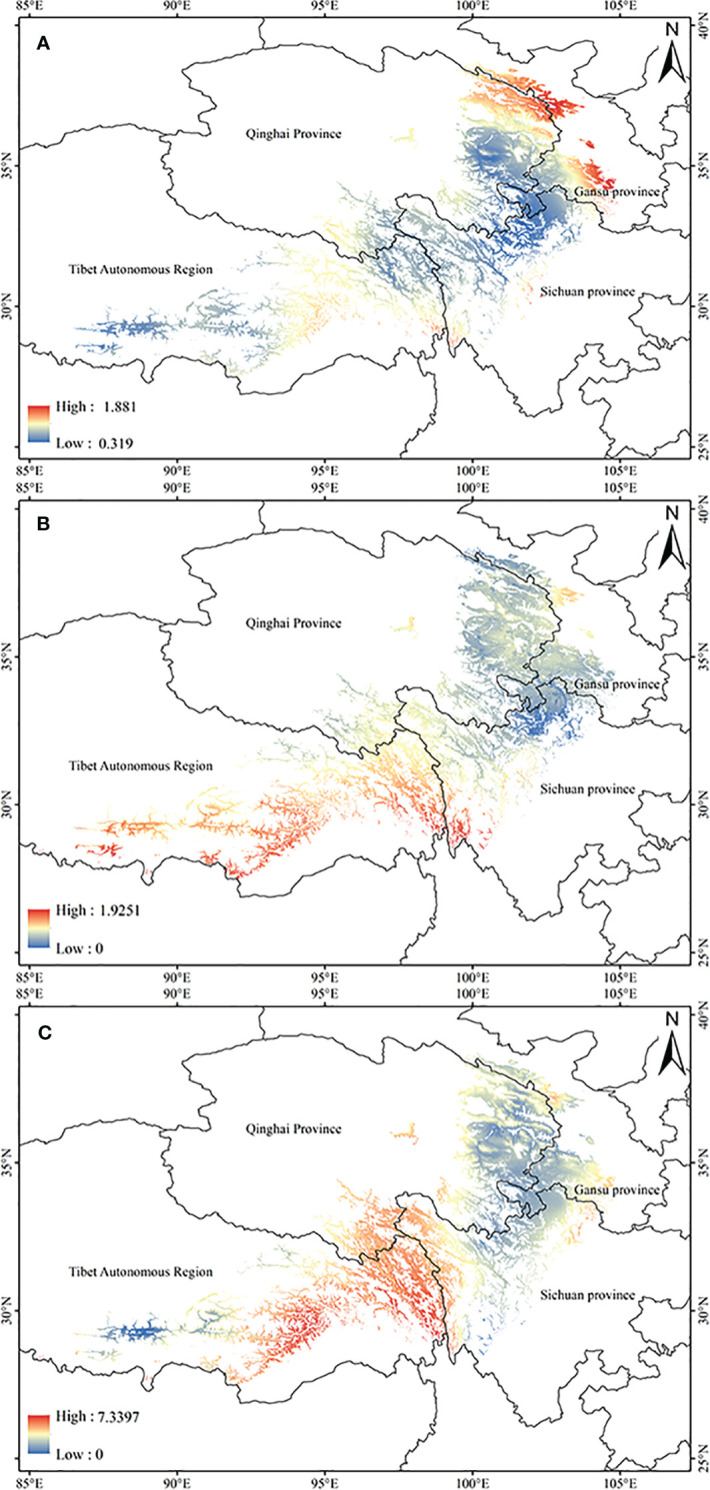
Regional suitability maps of high-quality of *A*. *tanguticus* under the current climate [**(A)** distribution pattern of anisodamine; **(B)** distribution pattern of anisodine; **(C)** atropine].

Based on the relationship model and the data of ecological factor layers, the ArcGIS was used to estimate the tropine alkaloids content spatial distribution of *A. tanguticus* in the suitable area in contemporary (**Figure 3**). The results indicate that the content of anisodamine in *A. tanguticus* is relatively higher in the Qilian Mountain area and Gannan city (Gansu Province), with eastern Tibet following suit (**Figure 3A**). The anisodine content in *A. tanguticus* decreases gradually from south to north, with relatively higher levels observed in Tibet (**Figure 3B**). Conversely, the atropine content in *A. tanguticus* peaks in the Hengduan Mountains and diminishes toward the north and west, with them as the center (**Figure 3C**).

**Figure 4:**
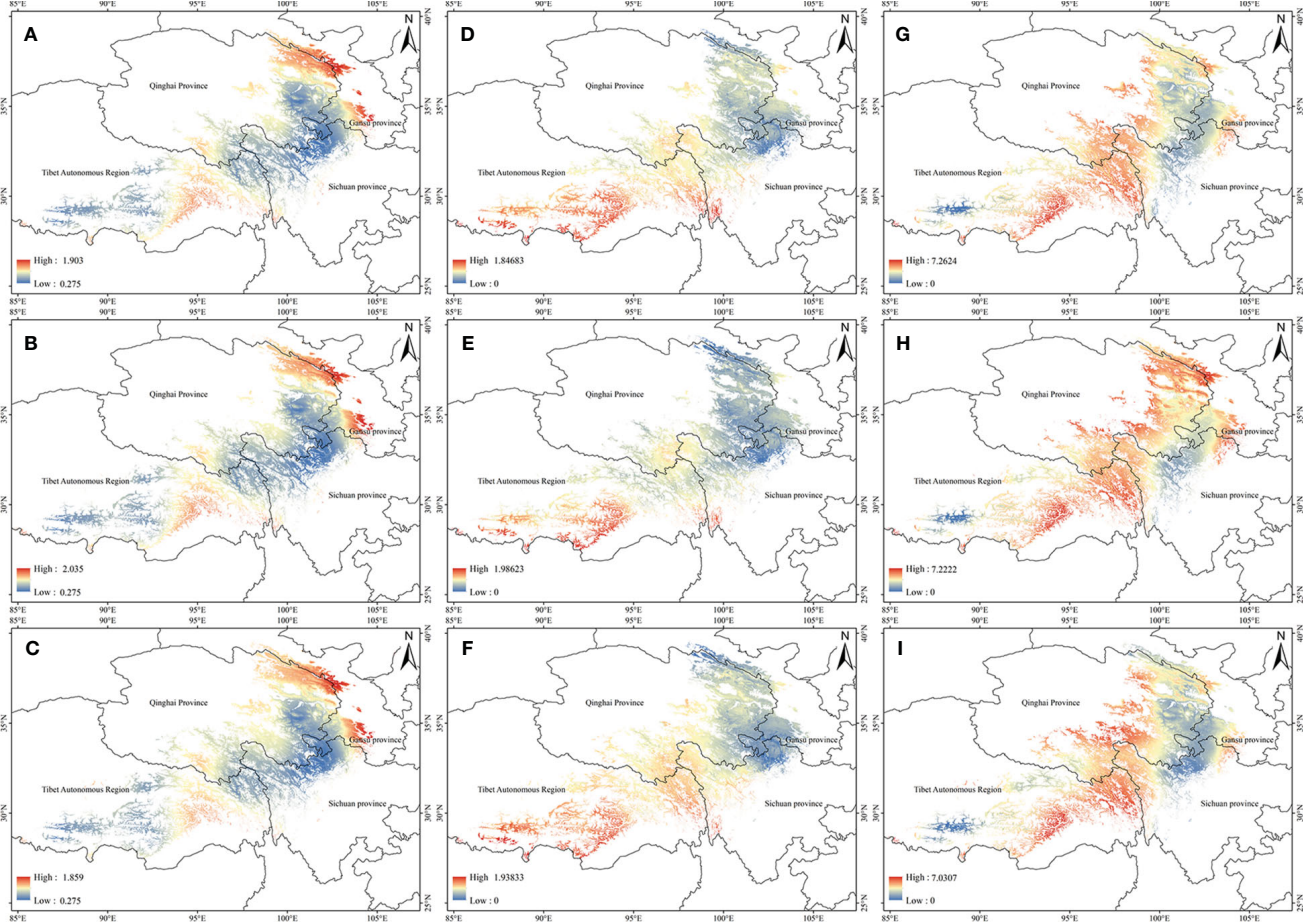
Regional suitability maps of high-quality of *A*. *tanguticus* under three climatic scenarios in the 2050s [**(A)** distribution pattern of anisodamine under RCP2.6 scenario; **(B)** distribution pattern of anisodamine under RCP6.0 scenario; **(C)** distribution pattern of anisodamine under RCP8.5 scenario; **(D)** distribution pattern of anisodine under RCP2.6 scenario; **(E)** distribution pattern of anisodine under RCP6.0 scenario; **(F)** distribution pattern of anisodine under RCP8.5 scenario; **(G)** distribution pattern of atropine under RCP2.6 scenario; **(H)** distribution pattern of atropine under RCP6.0 scenario; **(I)** distribution pattern of atropine under RCP8.5 scenario].

**Figure 5:**
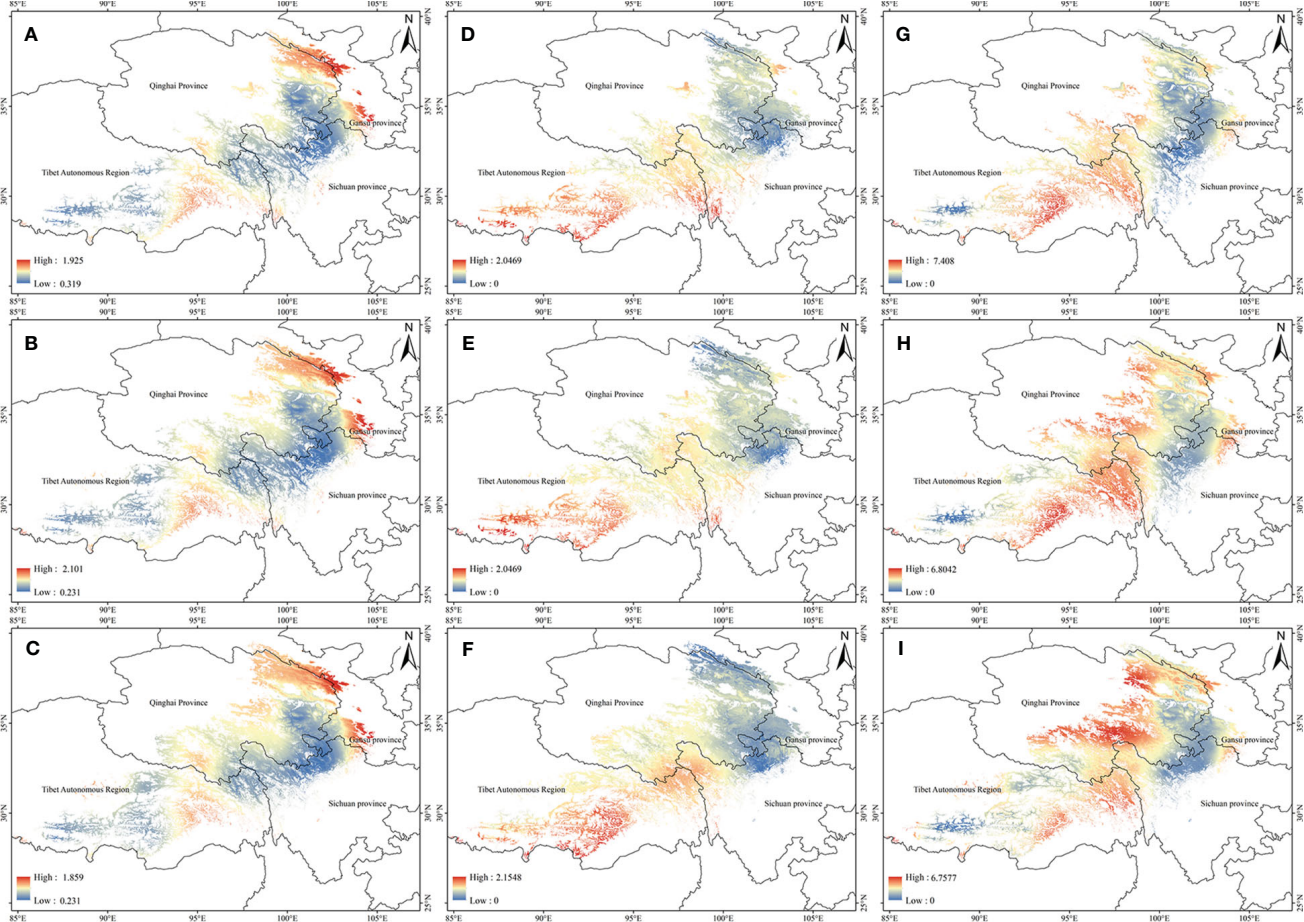
Regional suitability maps of high-quality of *A*. *tanguticus* under three climatic scenarios in the 2070s [**(A)** distribution pattern of anisodamine under RCP2.6 scenario; **(B)** distribution pattern of anisodamine under RCP6.0 scenario; **(C)** distribution pattern of anisodamine under RCP8.5 scenario; **(D)** distribution pattern of anisodine under RCP2.6 scenario; **(E)** distribution pattern of anisodine under RCP6.0 scenario; **(F)** distribution pattern of anisodine under RCP8.5 scenario; **(G)** distribution pattern of atropine under RCP2.6 scenario; **(H)** distribution pattern of atropine under RCP6.0 scenario; **(I)** distribution pattern of atropine under RCP8.5 scenario].

The potential high-quality suitable distribution areas under three climate change scenarios in the 2050s are shown in **Figure 4**. With the increase of the year, the quality area of anisodamine and anisodine in *A. tanguticus* remained unchanged, and the quality area of atropine showed an increasing trend. The potential distribution areas of high quality under three climate change scenarios scenario in the 2070s are illustrated in **Figure 5**. Over time, the quality area of anisodamine and anisodine in *A. tanguticus* diminished in Tibet and expanded in Qinghai. Conversely, the quality area of atropine exhibited a decreasing trend. Over the years, the quality area of anisodamine and atropine in *A. tanguticus* diminished in Tibet while expanding in Qinghai. Simultaneously, the quality area of anisodine increased in Qinghai.

## 4 Discussion

### 4.1 MaxEnt Model

The MaxEnt Model is a prediction method that makes unbiased inferences of unknown distribution based on limited known information. The model can still obtain satisfactory results, especially when the species distribution data is incomplete. Since 2010, the MaxEnt Model has been used in the suitability habitat and prediction of medicinal plant species distribution (Halvorsen et al., 2015; Ma et al., 2017; Dai et al., 2022; Zhao et al., 2023). This study collects the distribution sites of *A. tanguticus* with precise latitude and longitude combined with GIS technology and MaxEnt to make ecological suitability distribution predictions. **Figure 1** illustrates that the wild distribution area generated by ArcGIS software effectively encompasses nearly all the distribution sites and potential distribution areas of *A. tanguticus*. This indicates that the MaxEnt method, in conjunction with GIS technology, accurately predicts the ecological distribution area, offering a crucial reference for further elucidating the distribution areas of *A. tanguticus*.

### 4.2 Environmental factors on *A. tanguticus* distribution

This study analyzed the relationships between *A. tanguticus* and key environmental variables, and the corresponding response curves were obtained. The top four important factors affecting the distribution of *A. tanguticus* were Alt, Bio18, Bio 1, and Bio 7.

Altitude is an important ecological factor affecting the distribution pattern of biodiversity. This influence is typically driven by alterations in the hydrothermal conditions of habitats resulting from altitude increases. Temperature is the key factor for plant growth and development, physiological activities, and biochemical reactions (Geissler et al., 2019; Klem et al., 2019; Li et al., 2023). Bio 1 is the average annual temperature, which reflects the steady-state state of temperature and is the most used in daily life and climate change research (Chen et al., 2022a). In this study, when Bio 1 is between 0°C and 4.5°C, it is suitable for the growth and distribution of *A. tanguticus*. Bio 7 is the range of annual temperature changes representing the degree of cold winter and hot summer in a place. This parameter better captures the features of temperature change amplitude in a region. It can also indicate the degree to which the interaction between sea and land influences the climate of the region. In this study, when Bio 7 is between 33°C and 38°C, it is suitable for the growth and distribution of *A. tanguticus*, which is the way plants adapt to phenology. Therefore, the drastic temperature change in a period is not conducive to the survival of *A. tanguticus.* A suitable year temperature difference is conducive to plants using high temperature for photosynthesis, and using low temperature to weaken respiration and reduce the consumption of organic matter.

Precipitation is the most important ecological factor restricting growth and development and determines ecosystems’ structure and function. The warmest season is the peak period of growth of *A. tanguticus*, indicating that the precipitation in the growing period is very important for the growth. The suitable value range of the warmest season precipitation range (Bio 18) is 200~380 mm, ensuring adequate moisture without excessive humidity. This aligns with the growth environment of *A. tanguticus*, which is not tolerant to drought or flooding, as excess water content can easily lead to root rot. The suitable range of Bio15 (85~100), meaning different seasons have different water requirements, corresponding to the biological development characteristics of *A. tanguticus*., the plant ensures a long-term water supply due to the low germination potential during the greening period. After the greening period, it is necessary to reduce water to ensure root growth downward (Thioune et al., 2020; Zhang et al., 2021).

### 4.3 Environmental factors on *A. tanguticus* quality

Plant secondary metabolites are mean plant qualities that play an important role in plant growth and development, pathogen and animal defense, and biotic and abiotic stress (Wang et al., 2023). The environment can be directly or indirectly involved in the above processes by regulating plant secondary metabolites (Cheng et al., 2018; Zhang et al., 2023). In the predicted tropine alkaloid content equation, the monthly mean of the temperature difference between day and night (Bio 2) negatively effects on anisodamine content. Temperature seasonal variation variance (Bio 4), average annual precipitation (Bio 12), the wettest month precipitation (Bio 13), SOM, TN, and variance of seasonal variation in precipitation (Bio 15) are the most influential factor. Bio 4, Bio12, Bio15, and TN have a negative effect on anisodamine content, and Bio13 and SOM have a positive effect on anisodamine content. Average annual temperature (Bio 1), the wettest month precipitation (Bio 13), and variance of seasonal variation in precipitation (Bio 15) have a negative effect on atropine content, the monthly mean of the temperature difference between day and night (Bio 2), and the coldest month minimum temperature (Bio 6) have a positive effect on atropine content.

Drought stresses affect plant metabolism, leading to the closure of stomata and reduce photosynthesis. This, in turn, affects the synthesis and accumulation of secondary metabolites of plants (Rahimmalek et al., 2017; Cheng et al., 2018). Precipitation has a negative effect on tropine content, which confirms the above point. Temperature is a climatic factor that affects the synthesis of secondary metabolites, and its changes are not only naturally occurring but also influenced by climatic and seasonal changes (Szakiel et al., 2011). The increase or decrease of temperature will affect the synthesis and accumulation of plant secondary metabolism. For instance, *Scutellaria*, *Helichrysum*, and *Ocimum basilicum* exhibit an accumulation of compound content at low temperatures (Guo et al., 2013; Melito et al., 2016; Mostafavi et al., 2019). At the same time, *Scrophularia*, Aloe, and similar species demonstrate an accumulation of compound content at high temperatures (Yang et al., 2011; Kumar et al., 2017). In this study, the influence of temperature factors on tropine alkaloids has positive and negative effects, consistent with the previous research results.

The ecological environment profoundly impacts the growth, development, and synthesis of secondary metabolites in plants. Longitude, latitude, and altitude variations bring about distinct changes in environmental factors such as moisture, temperature, light intensity, and others. As altitude increases, the average annual temperature decreases, while CO_2_ concentration also declines. Furthermore, diurnal temperature variation and light intensity responses are heightened with altitude, affecting the maximum photosynthetic rate of plants and thereby shaping their growth distribution. High temperatures exert significant effects on plant internal moisture, photosynthesis, respiration, and mineral absorption.

Soil moisture, organic matter, and nutrients like nitrogen, phosphorus, and potassium serve as vital indicators of soil fertility. Insufficient soil fertility during plant growth may lead to growth impairments, developmental issues, susceptibility to pests and diseases, and diminished quality. Nitrogen and phosphorus, crucial nutrients for plant life processes, play essential roles in the composition of biological substances such as membranes, phospholipids, proteins, and nucleic acids. Plants commonly face limitations in nitrogen and phosphorus nutrients during growth, prompting adaptive changes in the morphological structure and biomass of underground plant parts in response to soil conditions.

The production of secondary metabolites, including tropane alkaloids in medicinal plants, constitutes a significant physiological metabolic process. The synthesis and accumulation of these compounds are intricately linked to the surrounding environment. Plants adjust the types and quantities of secondary metabolites based on environmental cues, with these compounds often contributing to plant resilience against adversity, diseases, pests, and other stressors. Various environmental factors, including population density, moisture, temperature, precipitation, soil properties, inorganic elements, and aspect, influence the accumulation of secondary metabolites. However, the effects of ecological factors on secondary metabolites are interactive and complex.

Moreover, the ecological environment directly affects plant metabolism, thereby influencing the synthesis of secondary metabolites such as tropane alkaloids. Warmer temperatures may accelerate metabolic rates, potentially affecting alkaloid production. Precipitation levels impact water availability, a critical factor for plant growth and metabolite synthesis, which can either enhance or limit metabolite production depending on species-specific water requirements and tolerance levels. Higher elevations, characterized by cooler temperatures and increased UV radiation, may stimulate the production of secondary metabolites as a protective response. The unique stress conditions at high altitudes could also lead to more pronounced differentiation in the quality of medicinal compounds. Additionally, the availability of essential nutrients like nitrogen, phosphorus, and potassium significantly influences plant health and metabolic activity, thereby shaping the biosynthesis of important medicinal compounds.

### 4.4 Impact of climate change on the suitable areas of *A. tanguticus*

In the present climatic conditions, the primary concentration of the suitable growing area for *A. tanguticus* is in the Eastern Tibetan Plateau, encompassing 3.21% of the total land area in China. As carbon emission concentrations rise, there is an observable increase in the distribution area and range of *A. tanguticus*. This suggests that future climate change significantly influences its potential distribution range and suitable habitat. The area of suitable areas will account for 4.21%, 4.22%, and 4.66% in 2050s, and 4.03%, 4.95%, and 5.40% in 2070s of Chinese land area under three RCPs. The range of suitable areas tends to extend westward and to high altitudes in 2050s and 2070s. With increased emission intensity, rainfall and temperature on the Qinghai-Tibet Plateau will increase. This indicates that warming in the high-altitude area in the northern part of the Qinghai-Tibet Plateau is greater than that in the low-altitude area in the southeast. Additionally, the most significant increase in precipitation is observed in the northwestern region (Chen et al., 2022e). With climate warming, species distribution will migrate to higher latitudes and higher altitude areas (Parmesan and Yohe, 2003; Zhong et al., 2004). The suitable area of *A. tanguticus* shows the same migration trend.

Our results show that suitable areas of *A. tanguticus* are distributed in the semi-humid and semi-arid areas in the Qinghai-Tibet Plateau of China. Recently, the Qinghai-Tibet Plateau has shown a humidity trend, and the entire region may continue to increase the humidity intensity in the next 30 years (Zhang et al., 2018). Therefore, the expansion area in Qinghai-Tibet Plateau may be related to this. By 2050s, 15%-37% of species will be “endangered” under moderate climate warming. Our results show that the suitable area of *A. tanguticus* shows an overall increasing trend under future climate conditions, indicating that there may be new areas suitable for growth in the future. This implies that *A. tanguticus* is not at risk of extinction due to climate change. However, overharvesting poses a risk of local disappearance for *A. tanguticus* due to its high economic value.

### 4.5 Practical application of predictions

*A. tanguticus* grows in Qinghai-Tibet Plateau and has harsh requirements for the growth environment. Hence, the cultivation site selection work is extremely important, which is a problem and bottleneck for artificial planting. This study employed the MaxEnt model to predict a map of suitable areas, providing valuable information for government agencies and farmers to make informed decisions regarding the cultivation. We have established an *A. tanguticus* cultivation base in Huanzhong District, Qinghai Province, China (36°47′7.08″N, 101°30′49.30″E, 2600 m) (**Supplementary Figure 4**). According to our study, this site located in the area predicted as the most suitable. The suitability of this cultivation area for *A. tanguticus* was confirmed by measuring both the morphology and tropine alkaloids content in the samples (Liu et al., 2022b; Chen et al., 2023). Therefore, the regions predicted in this study are likely to be good locations for developing an *A. tanguticus* cultivation industry.

## 5 Conclusions

This study used the MaxEnt model to simulate the spatial distribution of *A. tanguticus* suitable area and quality area under current and future climate change scenarios. The results indicated that among the environmental variables, Alt, Bio18, Bio 1, Bio 7, and AN play a crucial role in determining the spatial distribution of *A. tanguticus*. Under the current climate conditions, *A. tanguticus* suitable areas are mainly distributed in the Eastern Qing-Tibetan Plateau. Under future climate change, the suitable areas for *A. tanguticus* in China will be increased and the suitable areas show a high latitude shifting trend. Our results can provide useful references for species conservation, cultivation planning and sustainable utilization strategy of *A. tanguticus*.

## Data availability statement

The original contributions presented in the study are included in the article/**Supplementary Material**. Further inquiries can be directed to the corresponding author.

## Author contributions

CC: Funding acquisition, Writing – original draft. BW: Methodology, Writing – original draft. JL: Writing – review & editing. YX: Writing – original draft. KC: Writing – original draft. NL: Writing – review & editing. GZ: Supervision, Writing – review & editing.
