# Supplementary figures and images for: Predicting potential and quality distribution of Anisodus tanguticus (Maxim.) Pascher under different climatic conditions in the Qinghai–Tibet plateau
Source: Front Plant Sci. 2024 Jun 3;15:1369641. doi: 10.3389/fpls.2024.1369641 (PMC11180894; doi:10.3389/fpls.2024.1369641)

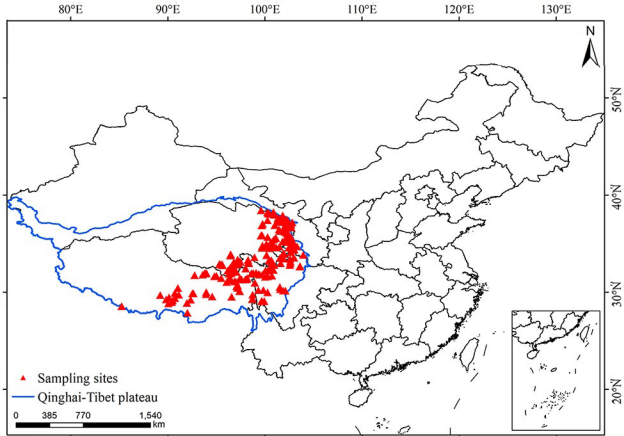

Supplement: Supplementary file 1 [file DataSheet_1.pdf]

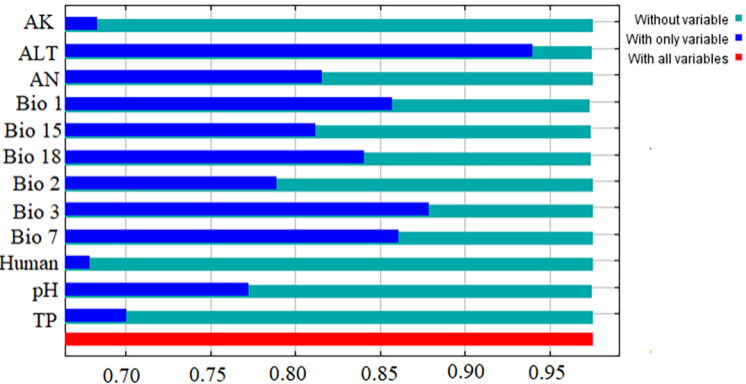

Supplement: Supplementary file 2 [file DataSheet_2.pdf]

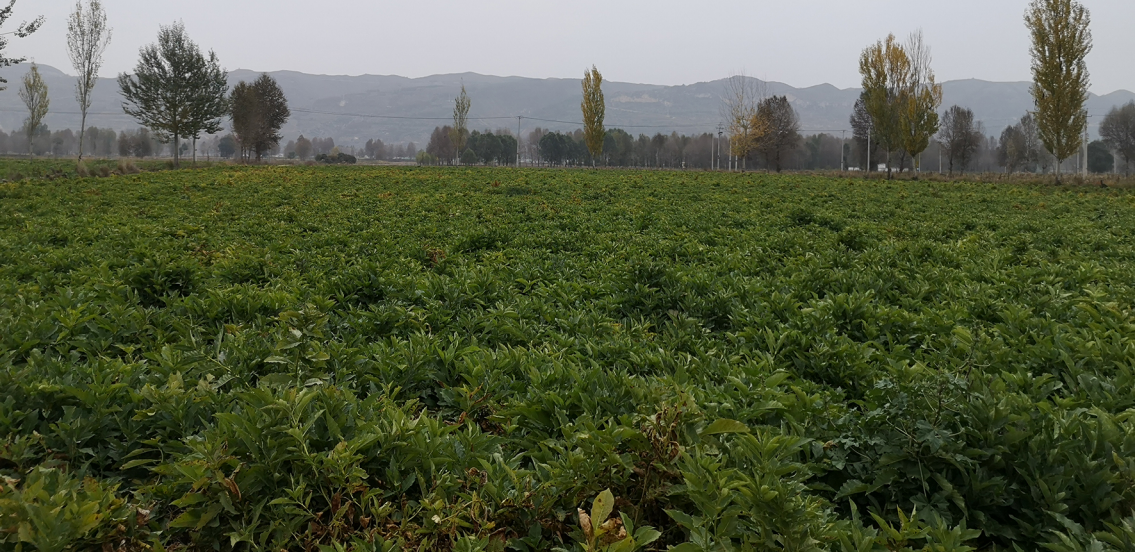

Supplement: Supplementary file 4 [file DataSheet_4.pdf]
