# Supplementary material for: Predicting potential and quality distribution of Anisodus tanguticus (Maxim.) Pascher under different climatic conditions in the Qinghai–Tibet plateau
Source: Front Plant Sci. 2024 Jun 3;15:1369641. doi: 10.3389/fpls.2024.1369641 (PMC11180894; doi:10.3389/fpls.2024.1369641)
Supplement: Supplementary file 3 [file DataSheet_3.pdf]

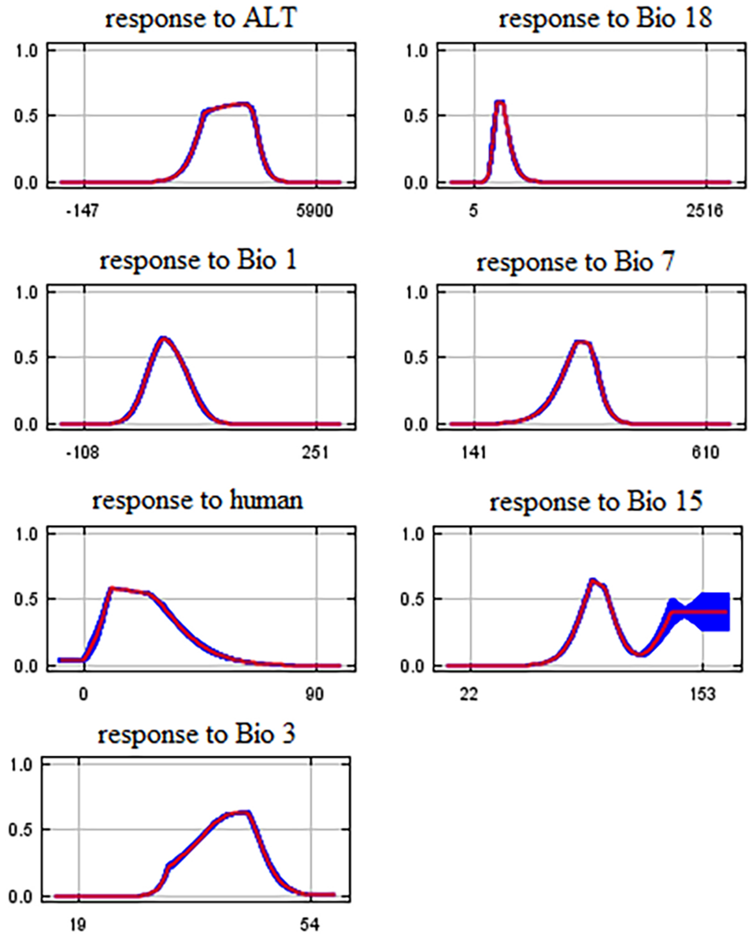
response to ALT
1.0
0.5
0.0
-147
5900
response to Bio 18
1.0
0.5
0.0
5
2516
response to Bio 1
1.0
0.5
0.0
-108
251
response to Bio 7
1.0
0.5
0.0
141
610
response to human
1.0
0.5
0.0
0
90
response to Bio 15
1.0
0.5
0.0
22
153
response to Bio 3
1.0
0.5
0.0
19
54
