# Supplementary material for: Predicting potential and quality distribution of Anisodus tanguticus (Maxim.) Pascher under different climatic conditions in the Qinghai–Tibet plateau
Source: Front Plant Sci. 2024 Jun 3;15:1369641. doi: 10.3389/fpls.2024.1369641 (PMC11180894; doi:10.3389/fpls.2024.1369641)
Supplement: Supplementary file 5 [file Table_1.docx]

**Predicting potential and quality distribution of *Anisodus tanguticus* (Maxim.) Pascher under different climatic conditions in the Qinghai–Tibet plateau**

**Chen Chen ^a, #^, Bo Wang ^b, #^, Jianan Li ^b^, Yuanming Xiao ^b^, Kaiyang Chen ^b^, Na Liu ^b^, Guoying Zhou ^b*^**

*^a^ Anhui Provincial Engineering Laboratory for Efficient Utilization of Featured Resource Plants, College of Life Sciences, Huaibei Normal University, Huaibei, Anhui 235000, PR China*

*^b^* *Chinese Academy of Sciences Key Laboratory of Tibetan Medicine Research, Northwest Institute of Plateau Biology, Xining, 810008, China*

^#^ Chen Chen and Bo Wang contributed equally to this manuscript

* Correspondence: Guoying Zhou, PhD, CAS Key Laboratory of Tibetan Medicine Research, Northwest Institute of Plateau Biology, 23^#^ Xinning Road, Xining, 810008, China. Email: zhougy@nwipb.cas.cn

Table S1 Percentage Contribution and Permutation Importance of environment variables for *A. tanguticus* in the Maxent

| Variable | Percent contribution | Permutation importance |
| --- | --- | --- |
| Alt | 59.6 | 61.7 |
| Bio 18 | 21.5 | 17.5 |
| Bio 1 | 6.7 | 12.3 |
| Bio 7 | 4.6 | 1.5 |
| human activities | 2.6 | 0.7 |
| Bio 15 | 1.6 | 2.7 |
| Bio 3 | 1.0 | 1.9 |
| AN | 1.0 | 0.3 |
| Bio 2 | 0.6 | 0.7 |
| pH | 0.4 | 0.2 |
| TP | 0.3 | 0.2 |
| AK | 0.2 | 0.3S |
